# Barriers and enablers to return to work after stroke in Maputo, Mozambique: A qualitative study

**DOI:** 10.4102/ajod.v15i0.2077

**Published:** 2026-08-19

**Authors:** Levy M.M.F. Pinto

**Affiliations:** 1 Department of Physical and Rehabilitation Medicine, Maputo Central Hospital, Maputo, Mozambique

**Keywords:** stroke, rehabilitation, return to work, disability, Mozambique

## Abstract

**Background:**

Stroke frequently results in reduced work participation or permanent withdrawal from work. In low- and middle-income countries like Mozambique, return to work is influenced by limited rehabilitation services, socioeconomic challenges and workplace factors. Little is known about stroke survivors’ experiences of these barriers and enablers in Mozambique.

**Objectives:**

To explore stroke survivors’ perceptions of barriers and enablers influencing return to work in Maputo, Mozambique.

**Method:**

A qualitative, exploratory, descriptive study was conducted using semi-structured face-to-face interviews with 18 purposively selected stroke survivors attending outpatient rehabilitation services at Maputo Central Hospital. Interviews explored participants’ experiences of returning to work after stroke and were audio-recorded, transcribed and analysed using thematic content analysis. Findings were interpreted using the International Classification of Functioning, Disability and Health (ICF).

**Results:**

Findings were organised according to the ICF framework. Barriers to return to work included impairments in body functions and structures (motor deficits, fatigue and cognitive difficulties), limitations in activities, restrictions in work participation and environmental factors such as limited vocational rehabilitation, inaccessible transport, workplace discrimination and inadequate employer support. Key enablers included functional gains through rehabilitation, family and social support, workplace accommodations and strong personal motivation to resume employment.

**Conclusion:**

Return to work after stroke is a multidimensional process shaped by interaction between body functions and structures, activities and participation, environmental and personal factors. Addressing these factors through comprehensive rehabilitation, vocational support, workplace accommodations and coordinated health and employment policies may improve sustainable work reintegration for stroke survivors in Mozambique.

**Contribution:**

This study highlights context-specific barriers and enablers to return to work after stroke in Mozambique, informing rehabilitation practice and disability-inclusive employment strategies.

## Introduction

Stroke is one of the leading causes of death and long-term disability worldwide, with more than 12 million new cases occurring annually and over 100 million people living with its long-term consequences (Feigin et al. 2021; Johnson et al. 2016). Although advances in acute stroke care have improved survival, many stroke survivors continue to experience persistent motor, cognitive, communication and emotional impairments that restrict their functioning and participation in daily life, including employment (Feigin et al. 2021; World Health Organization [WHO] 2022). The burden of stroke is particularly high in low- and middle-income countries (LMICs), which account for more than 80% of stroke-related deaths and disability, reflecting demographic transitions, increasing cardiovascular risk factors and limited access to comprehensive rehabilitation services (Owolabi et al. 2021).

Return to work (RTW) is increasingly recognised as an important indicator of successful stroke rehabilitation because it reflects not only physical recovery, but also social participation, economic independence and quality of life. Successful RTW remains challenging, with considerable variation across countries depending on the organisation of health systems, labour market characteristics and access to rehabilitation and vocational support services (Daniel et al. 2009; Edwards et al. 2018). Recent systematic reviews have shown that RTW is influenced by multiple interrelated factors, including motor impairment, cognitive dysfunction, post-stroke fatigue, depression, aphasia, job characteristics, family support, workplace accommodations and employer attitudes (Orange et al. 2024; La Torre et al. 2022). Similarly, evidence suggests that vocational rehabilitation and workplace interventions may improve work reintegration, although their availability remains limited in many settings (Pearce et al. 2023).

The International Classification of Functioning, Disability and Health (ICF) provides a comprehensive biopsychosocial framework for understanding functioning, disability and participation (WHO 2001). Rather than considering disability solely as a consequence of disease, the ICF conceptualises functioning as the dynamic interaction between body functions and structures, activities, participation, environmental factors and personal factors. Within this framework, RTW is regarded as a participation outcome influenced not only by neurological recovery, but also by contextual factors, including access to rehabilitation, family support, workplace adaptations, transportation, social attitudes and vocational opportunities. Consequently, the ICF offers an appropriate conceptual framework for exploring the complex experiences of stroke survivors returning to work.

In Mozambique, stroke represents a major public health challenge and remains an important cause of adult disability (Damasceno et al. 2010). Rehabilitation services are available in secondary- and tertiary-level hospitals and are provided predominantly on an outpatient basis. However, the country currently has no specialised inpatient rehabilitation units dedicated to multidisciplinary stroke rehabilitation. Following discharge from acute medical wards, patients usually continue rehabilitation through outpatient services when these are available, while structured vocational rehabilitation and coordinated RTW programmes are largely absent. The Strategic Plan for Physical and Rehabilitation Medicine 2023–2030 further identifies important challenges related to rehabilitation workforce capacity, continuity of care, access to assistive technology, community-based rehabilitation and service organisation, all of which may influence functional recovery, community participation and work reintegration (Ministry of Health of Mozambique 2023).

Although research on RTW after stroke has expanded considerably during the last decade, most evidence originates from high-income countries with well-established rehabilitation systems. Little is known about the experiences of stroke survivors living in LMICs, where health systems, labour markets and rehabilitation services differ substantially. To our knowledge, no qualitative study has specifically explored the perceived barriers and enablers to RTW among stroke survivors in Mozambique. Understanding these experiences is essential for informing rehabilitation practice, strengthening vocational rehabilitation services and guiding disability-inclusive policies aimed at improving work participation and social inclusion. Therefore, this study aimed to explore the perceived barriers and enablers to return to work among stroke survivors in Maputo, Mozambique, using the ICF as the conceptual framework for interpreting participants’ experiences.

## Research methods and design

### Study design

This study adopted a qualitative, exploratory and descriptive design. A qualitative approach was considered appropriate because the study sought to explore stroke survivors’ perceptions and experiences regarding barriers and enablers to return to work within their real-life context. Given the limited evidence from Mozambique and other low-income settings, an exploratory design enabled an in-depth understanding of participants’ lived experiences, while the descriptive component facilitated a comprehensive account of the personal, social, environmental and health system factors influencing return to work. The study was guided by the ICF, which informed both data collection and interpretation of findings.

### Setting

The study was conducted at the Physical and Rehabilitation Medicine services of Maputo Central Hospital. The department provides multidisciplinary rehabilitation services for adults and children with neurological, musculoskeletal and other disabling conditions and receives referrals from Maputo City, throughout Maputo Province, and other provinces across Mozambique.

Stroke survivors typically begin rehabilitation during the acute phase while admitted to hospital wards. Following discharge, they continue rehabilitation on an outpatient basis after assessment by a Physical and Rehabilitation Medicine specialist. At the time of the study, there were no specialised inpatient rehabilitation services or structured vocational rehabilitation programmes available at the study institution or elsewhere in Mozambique. Consequently, issues related to return to work were addressed within routine outpatient rehabilitation and community reintegration, without formal vocational rehabilitation pathways or structured return-to-work programmes.

### Participants

Eighteen stroke survivors were purposively selected to obtain diverse perspectives regarding return to work after stroke. Purposive sampling was used to maximise variation in age, sex, educational level, occupation before stroke and employment status after stroke, thereby enhancing the richness of the data.

Participants were eligible if they had a confirmed diagnosis of stroke documented in their medical records; were aged between 18 and 65 years; had been employed or self-employed before the stroke; were residing in the cities of Maputo or Matola; were medically stable and attending outpatient rehabilitation; and were able to communicate sufficiently to participate in an in-depth interview.

The ability to communicate was determined through clinical assessment conducted by the rehabilitation physician during routine outpatient consultations. Participants were required to demonstrate adequate comprehension of interview questions and the ability to express their experiences verbally in Portuguese. Individuals presenting severe aphasia, severe cognitive impairment or communication deficits that prevented meaningful participation were excluded from the study.

Sampling continued until data saturation was achieved, whereby no new themes emerged from successive interviews.

### Data collection

Data were collected through individual semi-structured interviews conducted between November and December 2022 by the first author, a clinician in Physical and Rehabilitation Medicine with experience in stroke rehabilitation. A semi-structured interview guide was developed based on the study objectives, relevant literature and the ICF framework. The guide included open-ended questions exploring participants’ experiences of returning to work, perceived barriers, enablers and suggestions for improving vocational reintegration.

Interviews were conducted in a private room within the rehabilitation department to ensure confidentiality and minimise interruptions. Each interview lasted approximately 45 min and was conducted in the participant’s preferred language (Portuguese or Changana). When necessary, participants were encouraged to clarify or elaborate on their responses to enhance the depth and richness of the data.

With participants’ written informed consent, all interviews were audio-recorded and subsequently transcribed verbatim. Interviews conducted in Changana were translated into Portuguese during transcription, with careful attention to preserving the original meaning of participants’ accounts. Field notes were recorded during and immediately after each interview to document non-verbal communication, contextual observations and the researcher’s reflexive notes, thereby supporting subsequent data interpretation. No repeat interviews were conducted, as the information obtained was considered sufficient to address the study objectives.

### Data analysis

The interview transcripts were analysed using thematic analysis following the six-phase approach described by Braun and Clarke (2006). Data analysis began immediately after the first interviews and continued concurrently with data collection, allowing emerging findings to inform subsequent interviews and facilitating the identification of data saturation.

Initially, the transcripts were read repeatedly to achieve familiarisation with the data. Meaningful units of text were then coded inductively, and similar codes were grouped into preliminary categories. Through an iterative process of reviewing and refining the codes and categories, broader themes were developed to capture participants’ experiences of returning to work after stroke.

Following theme development, the findings were interpreted using the ICF (WHO 2001) as the conceptual framework. Themes were mapped to the relevant ICF components – body functions and structures, activities and participation, environmental factors and personal factors – to facilitate interpretation of the multidimensional factors influencing return to work and the interaction between functioning and contextual factors.

Coding, theme development and interpretation were undertaken by the first author through repeated review of transcripts and codes to ensure consistency, credibility and analytical rigour. Data saturation was considered to have been achieved when successive interviews yielded no new codes or themes relevant to the study objectives.

### Researcher reflexivity

The interviews were conducted by the first author, a clinician in Physical and Rehabilitation Medicine with experience in stroke rehabilitation. Although this professional background facilitated an understanding of participants’ experiences and the clinical context, it also had the potential to influence data collection and interpretation. To minimise potential bias, the first author used a semi-structured interview guide consistently across all interviews, encouraged participants to express their views freely and adopted a reflexive approach throughout the research process by continuously reflecting on personal assumptions during data collection, coding and interpretation. Participants were informed that their decision to participate or decline would not affect the care they received.

### Ethical considerations

Ethical clearance to conduct this study was obtained from the Faculty of Medicine Maputo Central Hospital Institutional Health Bioethics Committee (No. CIBS FM&HCM/082/2022). Permission to conduct the study was also obtained from the management of the study site. All eligible participants received verbal and written information about the study and provided written informed consent before participating. Confidentiality was maintained by removing personal identifiers from transcripts and reports, assigning unique identification codes to participants and securely storing audio recordings and transcripts with restricted access to the research team.

## Results

The findings were organised according to the ICF, which conceptualises functioning as the dynamic interaction between impairments in body functions and structures, activity limitations, participation restrictions, environmental factors and personal factors (WHO 2001). Four interrelated themes emerged: (1) body functions and structures; (2) activities and participation; (3) environmental factors; and (4) personal factors. Within these components, participants described both barriers and enablers influencing their return to work.

### Body functions and structures

Within this ICF component, participants described barriers related to impairments in body functions, particularly motor and mental (cognitive) functions.

Motor impairments were among the most frequently reported barriers to returning to work. Fifteen of the 18 participants described persistent muscle weakness, impaired hand function and reduced mobility, which limited their ability to perform their pre-stroke occupational tasks. These impairments affected manual work, prolonged standing, walking and handling work equipment.

One participant stated:

‘I cannot use my hand properly like before, so I struggle to perform my tasks at work’. (P04, female, 39 years old, accountant)

Another participant stated:

‘Before the stroke I could carry heavy materials, but now I become tired very quickly and cannot do the same work’. (P06, male, 47 years old, mechanic)

A third participant stated:

‘Walking long distances is difficult for me, and my work requires me to move around all day’. (P16, male, 46 years old, sales representative)

Together, these accounts demonstrate that persistent motor impairments substantially reduced participants’ functional capacity, confidence and ability to resume their previous occupations.

Cognitive impairments also emerged as important barriers within this ICF component. Nine of the 18 participants described memory difficulties, reduced concentration and mental fatigue as factors that affected their work performance and their ability to perform tasks requiring sustained attention, planning and decision-making. These impairments reduced productivity and increased uncertainty about returning to employment.

One participant stated:

‘I forget things easily now, and I get tired quickly when trying to concentrate’. (P08, female, 49 years old, lawyer)

Another participant stated:

‘Sometimes I know what I want to do, but I lose my concentration and cannot finish the task’. (P01, male, 35 years old, business owner)

A third participant stated:

‘My memory is not the same as before. I need more time to remember things, and this makes it difficult to do my work’. (P04, female, 39 years old, accountant)

Overall, participants perceived residual motor and cognitive impairments as major barriers to work reintegration because these impairments limited their capacity to perform the physical and cognitive requirements of their previous work.

### Activities and participation

Within this ICF component, participants described both barriers and enablers related to their ability to resume work and participate in employment.

Participants reported considerable difficulties in performing work-related activities and resuming their previous occupational roles. Although some had regained independence in activities of daily living, 13 of the 18 participants reported that they remained unable to meet the physical and cognitive demands of their pre-stroke occupations. These activity limitations delayed or prevented their return to employment.

One participant stated:

‘I can take care of myself at home, but I still cannot do the work I used to do before the stroke’. (P14, male, 48 years old, security guard)

Another participant stated:

‘I tried to go back to work, but I realised I could not keep up with the pace that my job required’. (P09, male, 48 years old, teacher)

All 18 participants reported that participation in rehabilitation programmes improved their functional independence and ability to perform daily and work-related activities, thereby increasing their readiness to consider returning to work.

One participant stated:

‘The exercises helped me to become more independent, but I still needed guidance on how to return to my job’. (P12, female, 45 years old, bank employee)

Another participant stated:

‘After rehabilitation, I was able to do more things on my own, but returning to work was still difficult’. (P07, male, 41 years old, business owner)

Overall, participants described rehabilitation as an important enabler of activity performance and work participation. However, improvements in functional ability did not necessarily translate into a successful return to employment.

### Environmental factors

Within this ICF component, participants described both barriers and enablers related to the physical, social and organisational environments that influenced their return to work.

Environmental barriers included workplace discrimination, limited employer confidence, lack of task modification, architectural barriers and inadequate transportation. Participants explained that these factors restricted their opportunities to resume employment despite improvements in their physical condition.

The absence of structured vocational rehabilitation services was identified by 13 of the 18 participants as a major barrier to returning to work. Participants reported that rehabilitation services focused primarily on physical recovery, with limited preparation for returning to work, adapting to workplace demands or liaising with employers.

One participant stated:

‘I was taught how to walk again, but nobody talked to me about how I could go back to work’. (P16, male, 46 years old, sales representative)

Another participant stated:

‘When I finished rehabilitation, I still did not know whether I would be able to return to my job’. (P15, male, 54 years old, business owner)

Conversely, participants identified several environmental enablers that supported their return to work. Strong family support was identified by 10 of the 18 participants as an important enabler of return to work. Supportive employers were reported by seven of the 18 participants, flexible working arrangements by eight of the 18 participants, and encouragement from colleagues by five of the 18 participants. Together, these environmental supports facilitated work reintegration by promoting workplace inclusion, increasing participants’ confidence and encouraging sustained engagement in employment.

One participant stated:

‘My family encouraged me a lot, and that gave me strength to try working again’. (P10, male, 44 years old, administrative worker)

Another participant stated:

‘My employer allowed me to return gradually and adjusted some of my duties, which made a big difference’. (P13, male, 50 years old, shop assistant)

Overall, participants perceived that supportive environmental factors played a crucial role in facilitating return to work, whereas inaccessible environments, limited workplace accommodations and the absence of vocational rehabilitation reduced opportunities for successful work reintegration.

### Personal factors

Within this ICF component, participants described both barriers and enablers related to their personal perceptions, emotions and attitudes towards returning to work.

Fear of returning to work and reduced self-confidence were identified by 11 of the 18 participants as major personal barriers to work reintegration. Furthermore, 14 of the 18 participants questioned whether they would be able to meet the physical and cognitive demands of their previous jobs and expressed concerns about their ability to perform effectively in the workplace. These concerns reduced their confidence and willingness to attempt to return to employment.

One participant stated:

‘I am afraid to go back to work because I feel I am not the same person anymore’. (P10, male, 44 years old, administrative staff member)

Another participant stated:

‘Even if I wanted to return, I am not sure I can do my job like before’. (P11, female, 51 years old, field worker)

A third participant stated:

‘I worry that my employer will think I am no longer capable’. (P14, male, 48 years old, security guard)

Conversely, 10 of the 18 participants identified personal motivation, determination and hope as important enablers of return to work. They explained that returning to work represented not only financial security, but also an opportunity to regain independence, restore their sense of purpose and re-establish their social role. Despite persistent physical and cognitive impairments, these personal attributes encouraged them to continue rehabilitation and pursue work reintegration.

One participant stated:

‘I wanted to work again because I wanted to be independent and support my family’. (P02, male, 51 years old, business owner)

Another participant stated:

‘I kept believing that I would improve, and that hope encouraged me to keep trying’. (P06, male, 47 years old, mechanic)

A third participant stated:

‘Returning to work meant that I could feel useful again and regain my confidence’. (P12, female, 45 years old, bank employee)

Overall, participants’ narratives suggest that personal factors played a pivotal role in shaping return-to-work outcomes. While fear, uncertainty and reduced self-confidence discouraged work reintegration, motivation, determination and hope encouraged participants to pursue employment despite persistent challenges.

## Discussion

This study explored the perceived barriers and enablers to return to work among stroke survivors in Maputo, Mozambique, using the ICF as the conceptual framework. Overall, the findings demonstrate that return to work is a multidimensional process resulting from the interaction between impairments in body functions, activity limitations, participation restrictions, environmental factors and personal factors, rather than being determined solely by neurological recovery. These findings support the biopsychosocial perspective of the ICF, which conceptualises functioning as a dynamic interaction between health conditions and contextual factors (WHO 2001). They also reinforce growing evidence that successful work reintegration requires rehabilitation approaches extending beyond physical recovery to address occupational participation and social inclusion (Edwards et al. 2018; Nuccio et al. 2024; Owolabi et al. 2021; Pearce et al. 2023).

Although many barriers identified in this study are similar to findings reported in other low- and middle-income countries, contextual factors specific to Mozambique, including limited rehabilitation services, transportation challenges and labour market conditions, may further intensify difficulties in returning to work after stroke. These contextual characteristics should therefore be considered when interpreting the findings and designing rehabilitation and vocational reintegration strategies.

Within the ICF component of body functions and structures, residual motor and cognitive impairments emerged as important barriers to return to work. Muscle weakness, impaired hand function, reduced mobility, memory problems, reduced concentration and mental fatigue limited participants’ capacity to meet the physical and cognitive demands of their previous occupations. Although these impairments have consistently been identified as predictors of poor return-to-work outcomes (Barker-Collo et al. 2010; Orange et al. 2024; Westerlind, Persson & Sunnerhagen 2017), their impact appeared particularly pronounced in this study because many participants had previously been employed in physically demanding occupations or informal work, where opportunities for task modification and workplace accommodation are often limited. Consequently, even relatively mild residual impairments were perceived as sufficient to prevent work resumption. These findings emphasise that neurological recovery alone may not adequately reflect an individual’s ability to resume productive employment, particularly in low-resource settings where occupational demands are predominantly manual.

An important finding within the activities and participation component was that several participants had regained independence in activities of daily living but remained unable to resume their previous occupational roles. This distinction illustrates a fundamental principle of the ICF: recovery of activity performance does not necessarily translate into successful participation. Employment requires sustained physical effort, cognitive efficiency, social interaction and the ability to cope with workplace demands that extend beyond the requirements of basic daily activities. Similar observations have been reported in other studies, which demonstrate that functional independence is not, by itself, a reliable predictor of successful return to work after stroke (Ntsiea et al. 2015; Pearce et al. 2023; Wolf, Baum & Connor 2009). These findings therefore suggest that rehabilitation programmes should explicitly target work-related activities and occupational participation rather than focusing exclusively on restoring independence in daily living. This interpretation is consistent with recent evidence indicating that structured vocational rehabilitation and return-to-work interventions improve work participation after stroke (Li et al. 2024).

Environmental factors emerged as some of the most influential determinants of return to work. Participants consistently identified the absence of structured vocational rehabilitation services, limited workplace adaptations, employer-related barriers, inadequate transportation and architectural obstacles as major challenges to work reintegration. Conversely, strong family support, supportive employers, flexible working arrangements and encouragement from colleagues facilitated participants’ return to employment. These findings are similar to other studies demonstrating that environmental support frequently determines whether improvements in functional capacity translate into successful work participation (Andersen et al. 2012; Elloker & Rhoda 2018; Orange et al. 2024).

The findings also highlight an important contextual characteristic of rehabilitation in Mozambique. At the time of the study, rehabilitation services focused primarily on outpatient physical rehabilitation, with no specialised inpatient rehabilitation units or structured vocational rehabilitation programmes available. This reflects the current organisation of rehabilitation services in Mozambique, where stroke survivors typically receive rehabilitation during the acute hospital admission and continue rehabilitation on an outpatient basis following discharge, without access to specialised inpatient rehabilitation or formal vocational rehabilitation programmes. Recent evidence suggests that multidisciplinary vocational rehabilitation programmes, employer engagement and workplace-based interventions can facilitate successful return to work after stroke (Li et al. 2024). In contrast, participants in this study reported limited access to these forms of support. This service gap may partially explain why improvements in physical function did not consistently translate into successful return to work and underscores the importance of integrating vocational rehabilitation into existing rehabilitation services.

Personal factors also influenced participants’ experiences of returning to work. Fear of returning to work, reduced self-confidence and uncertainty regarding future work performance discouraged many participants from attempting to resume employment, despite improvements in physical functioning. Conversely, motivation, hope, determination and the desire to regain independence encouraged others to continue rehabilitation and pursue work reintegration. These findings reinforce previous evidence indicating that psychological readiness is an important determinant of work participation after stroke and may evolve independently of physical recovery (Coole, Radford & Grant 2013; Edwards et al. 2018; Medin, Barajas & Ekberg 2006). Together, these findings support the ICF perspective that personal factors interact with body functions, activities and environmental conditions to influence participation outcomes.

Taken together, the findings suggest that returning to work after stroke should be viewed as a complex rehabilitation outcome requiring coordinated interventions across multiple domains of functioning. Beyond economic productivity, successful return to work contributes to social participation, independence, personal identity and overall quality of life among stroke survivors (Vestling, Ramel & Iwarsson 2005). In the Mozambican context, strengthening rehabilitation services should therefore extend beyond improving physical recovery to include vocational rehabilitation, workplace assessments, employer engagement and community-based reintegration strategies. Integrating these components into existing rehabilitation services may improve long-term work participation, quality of life and social inclusion among stroke survivors. Given the increasing burden of stroke among working-age adults in low- and middle-income countries, these findings have important implications for rehabilitation policy and practice and highlight the need to incorporate return-to-work interventions into comprehensive stroke rehabilitation programmes.

### Strengths and limitations

This study provides in-depth insights into return-to-work experiences after stroke within the Mozambican context, using the ICF as a theoretical framework to explore the interaction between individual and contextual factors. The qualitative design enabled a rich understanding of participants’ experiences, which remain underrepresented in low-resource settings.

However, this study has several limitations. Firstly, participants were recruited from a single tertiary hospital in Maputo, which may limit the transferability of the findings to other regions of Mozambique, particularly rural settings. Secondly, the findings relied on self-reported experiences, which may be influenced by recall and social desirability bias. Thirdly, coding and thematic analysis were conducted by a single researcher. Although the coding process followed a systematic approach and themes were discussed within the research team, the absence of independent coding may have influenced data interpretation. Future qualitative studies should consider involving multiple coders or analyst triangulation to enhance the credibility, dependability and confirmability of the findings. Additionally, multicentre and longitudinal studies are recommended to better understand return-to-work trajectories over time.

## Conclusion

This study explored the perceived barriers and enablers to return to work among stroke survivors in Maputo, Mozambique, using the ICF as the conceptual framework. The findings demonstrate that return to work is influenced by the interaction between impairments in body functions, activity limitations, environmental conditions and personal factors, rather than by physical recovery alone.

Persistent motor and cognitive impairments limited participants’ ability to perform work-related activities, while fear of returning to work and reduced self-confidence further influenced work participation. At the same time, family support, supportive employers, flexible working arrangements and rehabilitation services were identified as important enablers of return to work. However, participants consistently highlighted the absence of structured vocational rehabilitation and workplace support as major barriers to successful work reintegration.

These findings provide important evidence from a low-resource setting where research on return to work after stroke remains scarce. They highlight the need for rehabilitation services in Mozambique to adopt a more comprehensive approach that integrates vocational rehabilitation, workplace engagement and community reintegration alongside conventional physical rehabilitation. Strengthening collaboration between the health, labour and social sectors may contribute to more sustainable return-to-work outcomes and improved social participation among stroke survivors.

The study also demonstrates the usefulness of the ICF as a framework for understanding the multidimensional nature of return to work after stroke and for guiding the development of person-centred rehabilitation programmes and policies in Mozambique and similar low- and middle-income countries.
